# The three-way relationship of polymorphisms of porcine genes encoding terminal complement components, their differential expression, and health-related phenotypes

**DOI:** 10.1186/1753-6561-5-S4-S19

**Published:** 2011-06-03

**Authors:** Klaus Wimmers, Do Vo Anh Khoa, Sabine Schütze, Eduard Murani, Siriluck Ponsuksili

**Affiliations:** 1 Leibniz Institute for Farm Animal Biology (FBN), 18196 Dummerstorf, Germany; 2 present address: Department of Animal Sciences, College of Agriculture and Applied Biology, Cantho University, 92100 Cantho City, Vietnam

## Abstract

**Background:**

The complement system is an evolutionary ancient mechanism that plays an essential role in innate immunity and contributes to the acquired immune response. Three modes of activation, known as classical, alternative and lectin pathway, lead to the initiation of a common terminal lytic pathway. The terminal complement components (TCCs: C6, C7, C8A, C8B, and C9) are encoded by the genes *C6*, *C7*, *C8A*, *C8B*, *C8G*, and *C9*. We aimed at experimentally testing the porcine genes encoding TCCs as candidate genes for immune competence and disease resistance by addressing the three-way relationship of genotype, health related phenotype, and mRNA expression.

**Results:**

Comparative sequencing of cDNAs of animals of the breeds German Landrace, Piétrain, Hampshire, Duroc, Vietnamese Potbelly Pig, and Berlin Miniature Pig (BMP) revealed 30 SNPs (21 in protein domains, 12 with AA exchange). The promoter regions (each ~1.5 kb upstream the transcription start sites) of *C6*, *C7*, *C8A*, *C8G*, and *C9* exhibited 29 SNPs. Significant effects of the TCC encoding genes on hemolytic complement activity were shown in a cross of Duroc and BMP after vaccination against Mycoplasma hyopneumoniae, Aujeszky disease virus and PRRSV by analysis of variance using repeated measures mixed models. Family based association tests (FBAT) confirmed the associations. The promoter SNPs were associated with the relative abundance of TCC transcripts obtained by real time RT-PCR of 311 liver samples of commercial slaughter pigs. Complement gene expression showed significant relationship with the prevalence of acute and chronic lung lesions.

**Conclusions:**

The analyses point to considerable variation of the porcine TCC genes and promote the genes as candidate genes for disease resistance.

## Background

Infectious diseases in farm animals and their control by therapeutic, prophylactic and metaphylactic treatments and veterinary management schemes cause immense costs and animal welfare concerns and increasingly burden man and environment with chemotherapeutics. Genetically improved health and resistance status of farm animals is associated with higher product quality, reduced danger of transmission of infectious agents or traces of antibiotics together with its positive impact on production costs and animal welfare. Breeding for specific and/or global disease resistance is hampered by the difficulty to discriminate the phenotypes `resistant' and `susceptible' either after experimental or natural infections. Alternatively, it has been proposed to measure immune response traits as indicator traits of improved defense power. However, determination of high and low immune responsiveness is still very inefficient and too complex for most farm animal breeding programs. Consequently, the availability of DNA-markers for disease resistance would be particularly useful in farm animal breeding. Since infectious factorial diseases occur in all porcine age groups and are especially responsible for economic losses and high mortality-rates in weaners and young feeder pigs affected by respiratory and enteritic diseases we focus on broad resistance against various pathogens.

The complement system is a mechanism of the innate immune system capable of direct killing of microorganisms and modulating phagocytosis, inflammation, humoral and cellular immune response. The complement system is a highly regulated and complex set of interacting proteins in blood plasma and on cells surfaces. There are three pathways of complement activation, the classical, the alternative and the lectin pathway. Activation of the complement system through the classical pathway depends on the presence of immune complexes and links the innate immune system to the adaptive. The alternative and lectin pathway represent major means of natural resistance to microorganisms in the absence of specific antibodies. All the three pathways lead to the initiation of a common terminal lytic pathway and the formation of the membrane attack complex (MAC), which causes lysis of the invading pathogens/microbes and is an assembly of the homologous components C5b, C6, C7, C8, and C9. The terminal complement components (TCCs) are encoded by the genes *C6*, *C7*, *C8A*, *C8B*, *C8G*, and *C9* originating from an ancestral gene. We have previously shown association of complement genes of the initiation pathways and other immune relevant genes with complement activity and acute phase response [1-4]. Here we aimed at structural and functional characterization of the porcine genes encoding TCCs and experimentally qualifying TCCs as candidate genes for immune competence and disease resistance by addressing the three-way relationship of genotype, health related phenotype, and mRNA expression.

## Methods

Experimental population: Animals (n=457) of an experimental F2 population based on reciprocal crossbreeding of Duroc and Berlin Miniature Pig (DUMI) [5] were immunized with Mycoplasma hyopneumoniae (Mh), Aujeszky disease (ADV), and PRRS (PRRSV) vaccines at 6, 14 and 16 weeks of age, respectively. Blood samples were taken immediately prior to immunisation (day 0) and at day 4 and 10 after Mh and ADV vaccination, but only at day 10 days after PRRSV vaccination. Hemolytic complement activities in the classical (CH50) and alternative pathways (AH50), C3c (C3c) and haptoglobin (HP) acute phase protein levels were measured [6]. The DUMI animals were genotyped at 88 loci (72 microsatellites, 16 biallelic markers) covering the porcine autosomes. Linkage analysis was performed using CRI MAP [7]. The QTL analysis was done using QTLexpress [8] under the line cross and the half sib model and after adjustment of phenotypic data for systematic effects by mixed models with and without repeated measures statement. Association and linkage of SNPs detected in the cDNA sequence of *C6*, *C7*, *C8A*, *C8B*, and *C9* were tested by means of analysis of variance using repeated measures mixed models and family based association test (FBAT) testing the null hypothesis of no association in the presence of linkage (option `eÂ´, computing the test statistic using the empirical variance [9]).

Commercial herds: Animals (n=311) of commercial herds of the breeds German Landrace, Large White and their crossbreeds (F1) and Piétrain × F1 were kept and performance tested at the Pig performance test station Jürgenstorf and slaughtered at the experimental abattoir of the Leibniz Institute of Farm Animals Biology (FBN). Liver tissue was immediately sampled after exsanguination for RNA isolation using Tri-Reagent (Sigma-Aldrich, Taufkirchen, Germany) and NucleoSpin RNA II kit (Macherey-Nagel, Düren, Germany) including DNase treatment. Lung lesions were recorded during veterinary inspection and relative abundance of transcripts of *C6*, *C7*, *C8A*, *C8B*, *C8G*, and *C9* was determined in liver tissue. Therefore, real time PCR was performed using the LightCycler 480 system (Roche, Mannheim, Germany) with cDNA synthesized from 1µg of total RNA using random and oligo d(T) 13VN primers, Superscript III reverse transcriptase (Invitrogen, Karlsruhe, Germany), and the LightCycler 480 SYBR Green I Master kit (Roche). Standard curves were generated by amplifying serial dilutions of specific PCR products. Normalization of variation in RT-PCR efficiency and initial RNA input was performed using the RPL32 and ALB genes as internal standards by dividing the calculated mRNA copy numbers by a mean normalization factor derived from the expression of the reference genes (relative abundance). Alternatively, ΔCT was calculated by subtracting the mean ct-values of the reference genes from the ct-values obtained for single TCC.

Association of SNPs detected in the promoters or cDNAs of *C6*, *C7*, *C8A*, and *C9* with relative transcript abundance were analyzed by mixed model analysis of variance (SAS, Proc mixed) taking into account the fixed effects of breed, farm where the piglets were born and raised until seven weeks of age when they were transported to the performance test station, genotype of the respective gene and the random effect of father. The relationship between the respective genotypes and the prevalence of lung lesions was analyzed by case control analysis using JMP genomics. The relationship between the transcript abundance and lung lesions was evaluated by analysis of variance taking breed and lung lesions (affected, non-affected) as fixed effects on the dependent variable of relative transcript abundance.

## Results and discussion

For genetic mapping of the TCCs and of QTL for complement activity and acute phase response as well as studying association and linkage of the complement genes animals of the experimental population DUMI were examined where multiple vaccinations and samplings were made. The QTL analysis for hemolytic complement activity in the classical and alternative pathway and acute phase response, i.e. C3c and HP serum concentrations, revealed 67 QTL with p < .05 genome-wide significance. The proximal region of SSC2 (orthologous to HSA11 0-70 Mb), the distal region of SSC4 (HSA1 95-155 Mb), and the intermediate region of SSC16 (HSA5 0-73 Mb & 150-174 Mb) showed several QTL clustered together [10]. In these regions a single true QTL might underlie the various QTL positions estimated for related traits. With regard to the number and the magnitude of their impact, QTL for humoral innate immune traits behave like those for other quantitative traits. Radiation hybrid and genetic mapping of complement genes (*C8G*: SSC1q2.13; *C8A* and *C8b*: SSC6q3.1-q3.5; *C6*, *C7*, *C9*: SSC 16q14) highlights them as positional candidate genes for the QTL [11].

In order to analyze association and linkage of the TCCs with complement activity the cDNA sequences were obtained and SNPs were detected by comparative sequencing of animals of the breeds German Landrace, Piétrain, Hampshire, Duroc, Vietnamese Potbelly Pig, and Berlin Miniature Pig (BMP). In total 30 SNPs were detected (21 in protein domains, 12 nonsynonymous) in the cDNA sequences of the genes *C6* (Acc. # DQ333199), *C7* (Acc. # AF162274; [12]), *C8A* (Acc. # DQ333200), *C8B* (Acc. # DQ333201), *C8G* (Acc. # DQ333202) and *C9* (Acc # DQ333198). Analysis of variance revealed association of *C7* (c.881A>G) and *C9* (c.407C>T) with complement activity at p<.05 and for *C8A* (c.1544C>T) at p=0.06 [13]. Family based association tests (FBAT) confirmed the associations (p<0.05). The results indicate considerable polymorphism of the coding region of porcine TCCs and promote the TCCs as candidate genes for innate humoral defense.

The analyses in the experimental F2 population allowed multiple vaccinations and samplings, mapping of QTL for complement activity and acute phase response as well as detection of association and linking of complement genes. However, in order to evaluate the results and to get closer to commercial conditions in terms of breeds and pedigrees - especially in view of the available SNP chips - and also diseases, further studies were focused on commercial herds, where relative transcript abundance of TCCs and lung lesions were used as traits related to health and the three-way relationship of genotype, health related phenotype, and mRNA expression was explored. Consequently, focus was on promoter regions for detection of further SNPs. The promoter regions (each ~1.5 kb upstream the transcription start sites) of *C6*, *C7*, *C8A*, *C8G*, and *C9* exhibited 29 SNPs. *In silico* analyses indicated that most of these SNPs were located in conserved regions or even in putative transcription factor binding sites. For association studies SNPs in the promoter of *C6* and *C9* (*C6*: g.-80C>G; *C9:* g.-737C>T) and in the cDNA-sequence of *C7* and *C8A* (*C7*: c.154A>G; *C8A:* c.1544C>T), respectively, were genotyped in 311 animals of commercial herds, of which relative abundance of transcripts of TCCs and lung lesion score were available. The minor allele frequencies obtained were ≥ 0.2. For *C7* (p=.05) and *C9* (p=.0003) association with relative transcript abundance was shown. Moreover, the analysis revealed interaction of polymorphisms and levels of expression among various complement genes. For *C9* significant differences of the genotype distribution among non-affected and affected animals was evident (p=.03). Moreover, the level of expression of *C7* (p=.02), *C8A* (p=.02), *C8B* (p=.09), and *C8G* (p=.05) differed between non-affected and affected animals [14].

## Conclusions

Due to the lack of trait records related to animal health from the field we proposed to evaluate transcript levels of immune genes including TCCs obtained at slaughter as biomarkers for disease resistance. Relationships between polymorphisms of the complement genes and their expression were apparent for several loci. Here, lung lesions, which were recorded during veterinary inspection, were used as parameters of health in order to get a first insight of any links between the polymorphisms, differential basal expression of TCCs, and disease resistance. For some TCCs this three-way relationship could be shown. Subsequent eQTL analyses and relating of traits relevant to health and performance will facilitate the identification of DNA-markers – which are the ultimate biomarkers for breeding purpose – for disease resistance. The results encourage further studies including functional assays of promoter SNPs, genome wide association studies for lung lesion prevalence and transcript abundance providing eQTL, monitoring the health status and sampling within commercial herds.

## Competing interests

The authors declare that they have no competing interests.

## Authors’ contributions

KW conceived and designed the study, contributed to data interpretation and drafted the manuscript; DVAK and SS contributed to obtaining sequences, SNPs and genotypes and helped in data analysis and drafting the manuscript; EM and SP managed phenotypic recording and sampling, aided in data analysis and drafting the manuscript.
